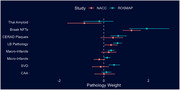# Estimating the total burden of multiple neuropathological changes

**DOI:** 10.1002/alz70860_100260

**Published:** 2025-12-23

**Authors:** Calum Alexander Hamilton, Stephen Wharton, Alan J Thomas

**Affiliations:** ^1^ Newcastle University, Newcastle upon Tyne, United Kingdom; ^2^ University of Sheffield, Sheffield, United Kingdom; ^3^ Newcastle University, Newcastle upon Tyne, Newcastle, United Kingdom

## Abstract

**Background:**

Multiple neuropathologies are known to contribute to dementia incidence during life. These may have a synergistic effect, with differently weighted contributions to cognitive progression.

Individual differences in overall multi‐pathological burden may account for the wide individual differences observed in the presence and progression of cognitive symptoms during later life, with some individuals being apparently cognitively healthy despite severe neuropathological changes, while some others present with dementia despite pathology not meeting diagnostic criteria.

Existing models for estimating this burden are limited by dichotomous analytical approaches. In this preliminary work, we aimed to estimate dose‐dependent contributions of multiple co‐pathologies to dementia using novel Bayesian methods in two independent cohorts.

**Method:**

This analysis used data from 9 ADRCs in the National Alzheimer's Coordinating Center (NACC) Neuropathological Uniform Data Set (funded by NIA/NIH Grant U24 AG072122), and the Religious Order Study/Rush Memory and Aging Project (ROSMAP) clinicopathological community cohort.

A novel Bayesian model was developed incorporating multiple neuropathological changes with non‐linear dose‐dependence: Alzheimer's disease related neuropathological changes (Thal Amyloid phase, Braak Neurofibrillary Tangle (NFT) stage, and CERAD neuritic plaque score), Lewy pathology, and cerebrovascular pathologies.

**Result:**

Complete multi‐pathological data were available from 1882 brain tissue donors in the development cohort, and 1007 in the validation cohort.

The development model identified strong total pathological contributions from NFT stage (β=1.9 [1.2 – 2.9]), neuritic plaque score (β=0.6 [0.3‐1.0]), Lewy pathology (β=0.6 [0.4‐0.8]), and macro‐infarcts (β=0.3 [0.2‐0.5]) which were externally validated.

Moderate contributions were also identified from cerebral small vessel disease and micro‐infarcts in the development cohort, but not externally validated.

The NACC case series had a higher baseline dementia risk in the absence of overt pathology in comparison to the ROSMAP community cohort.

**Conclusion:**

These analyses provide an initial validation that overall multi‐pathological burden may be estimated from community pathology cohorts. Estimated weights of key neuropathological changes were externally validated.

Future work will incorporate additional cohorts to refine estimated weights of microscopic infarcts and cerebral small vessel disease, incorporate uncommon neuropathological measures, and clarify the baseline risk of dementia in the absence of pathology.